# Real-time predictions of the 2018–2019 Ebola virus disease outbreak in the Democratic Republic of the Congo using Hawkes point process models

**DOI:** 10.1016/j.epidem.2019.100354

**Published:** 2019-07-23

**Authors:** J. Daniel Kelly, Junhyung Park, Ryan J. Harrigan, Nicole A. Hoff, Sarita D. Lee, Rae Wannier, Bernice Selo, Mathias Mossoko, Bathe Njoloko, Emile Okitolonda-Wemakoy, Placide Mbala-Kingebeni, George W. Rutherford, Thomas B. Smith, Steve Ahuka-Mundeke, Jean Jacques Muyembe-Tamfum, Anne W. Rimoin, Frederic Paik Schoenberg

**Affiliations:** a Department of Epidemiology and Biostatistics, University of California, San Francisco, CA, USA; b Department of Statistics, University of California, Los Angeles, CA, USA; c Center for Tropical Research, Institute of the Environment and Sustainability, University of California, Los Angeles, CA, USA; d Department of Epidemiology, University of California, Los Angeles, CA, USA; e Ministry of Health, Kinshasa, Congo; f School of Public Health, University of Kinshasa, Kinshasa, Congo; g Institut National de Recherche Biomedicale, Kinshasa, Congo; h F.I. Proctor Foundation, University of California, San Francisco, CA USA

**Keywords:** Ebola virus disease, Hawkes point process, Mathematical modelling, Democratic Republic of Congo, Compartmental models

## Abstract

As of June 16, 2019, an Ebola virus disease (EVD) outbreak has led to 2136 reported cases in the northeastern region of the Democratic Republic of the Congo (DRC). As this outbreak continues to threaten the lives and livelihoods of people already suffering from civil strife and armed conflict, relatively simple mathematical models and their short-term predictions have the potential to inform Ebola response efforts in real time. We applied recently developed non-parametrically estimated Hawkes point processes to model the expected cumulative case count using daily case counts from May 3, 2018, to June 16, 2019, initially reported by the Ministry of Health of DRC and later confirmed in World Health Organization situation reports. We generated probabilistic estimates of the ongoing EVD outbreak in DRC extending both before and after June 16, 2019, and evaluated their accuracy by comparing forecasted vs. actual outbreak sizes, out-of-sample log-likelihood scores and the error per day in the median forecast. The median estimated outbreak sizes for the prospective thee-, six-, and nine-week projections made using data up to June 16, 2019, were, respectively, 2317 (95% PI: 2222, 2464); 2440 (95% PI: 2250, 2790); and 2544 (95% PI: 2273, 3205). The nine-week projection experienced some degradation with a daily error in the median forecast of 6.73 cases, while the six- and three-week projections were more reliable, with corresponding errors of 4.96 and 4.85 cases per day, respectively. Our findings suggest the Hawkes point process may serve as an easily-applied statistical model to predict EVD outbreak trajectories in near real-time to better inform decision-making and resource allocation during Ebola response efforts.

## 1. Introduction

As of June 16, 2019, 2136 confirmed and probable cases of Ebola virus disease (EVD) were reported in North Kivu and Ituri Provinces of the Democratic Republic of the Congo (DRC) (WHO, 2019). Security issues resulting from activities of over 100 rebel and other insurgent groups, including attacks on Ebola treatment centers in Butembo and Katwa, have likely contributed to the ongoing nature of this EVD outbreak (Damon et al., 2018). Of the > 34 prior EVD outbreaks (CDC, 2019), none have occurred in a geographic region with a similar set of conflict issues. Moreover, the use of case counts from previous EVD outbreaks reported in the literature have proven unreliable in their ability to forecast an outbreak’s size (Worden et al., 2018; Asher, 2018). It is likely that additional time and effort will be required before all of the contributing factors to this outbreak can be properly assessed, parameterized, and modeled.

The Hawkes point process model, however, offers the Ebola modeling community a novel, rapid option to forecast outbreak size and spread (Meyer et al., 2012). Using modern methods, one can rapidly and nonparametrically estimate short-term outbreak size and rely on minimal modeling assumptions to do so (Schoenberg et al., 2017; Hawkes, 1971; Park et al., 2018). Decomposing peak history effects into the contribution of previous events and an average background rate, this point process model has long been used in the context of seismology to describe earthquakes and their aftershocks as well as other environmental science and biological phenomena (Hawkes, 1971; Gerhard et al., 2017; Schoenberg, 2004; Marsan and Lengliné, 2008). In some cases, Hawkes point process models have also been used to forecast the spatial and temporal spread of infectious disease outbreaks (Schoenberg et al., 2018; Meyer and Leonard, 2014; Meyer et al., 2012), including the 2013–2016 EVD outbreak in West Africa (Park et al., 2018).

There is an increasing body of evidence suggesting that short-term forecasts with few parameters are more reliable than long-term forecasts (particularly early in an outbreak) that determine the final outbreak size (Worden et al., 2018; Funk et al., 2018; Viboud et al., 2017; Chowell et al., 2017). In the context of an ongoing outbreak, many published statistical models have focused on long-term or final outbreak size (Meltzer et al., 2014; Kelly et al., 2018; Valdez et al., 2015; Chretien et al., 2015; Siettos et al., 2015). Given the advantages of the Hawkes model and the limitations of other statistical models in the ongoing EVD outbreak setting (Chowell et al., 2017), we fit the Hawkes point process model to daily EVD case counts to forecast case counts over subsequent weeks. It is our hope that this application of the Hawkes point process model may further engage outbreak responders on the value of short-term forecasts when making important public health decisions related to resource allocations.

## 2. Methods

Data were collected from the Ministry of Health and World Health Organization (WHO) situation reports on EVD case counts occurring in the northeastern region of DRC. The Ministry of Health initially released daily case counts while WHO situation reports confirmed these case counts with weekly reports (WHO, 2019). Our dataset included probable and confirmed EVD cases that occurred from the start of the outbreak on May 3, 2018, until June 16, 2019 (Supplement 1). (We only included in our models case counts from the EVD outbreak in the northeastern region of DRC. In 2018, there was another EVD outbreak that occurred in the western region of DRC, and WHO declared the end of this outbreak on July 24. Although there was a temporal overlap of the EVD outbreaks in DRC, they occurred approximately 1500 miles apart and there has been no evidence of an epidemiological or viral genetic link between them).

We fit the Hawkes point process model to daily EVD case counts reported in the northeastern region of DRC. Details of this estimation method can be found elsewhere (Park et al., 2018). Briefly, for point processes, the expected rate at which points (or cases) accumulate at time *t* is characterized by the conditional intensity λ*(t)*. Although versions of Hawkes models have parameters that describe these types of data in space and time, to be comparable with the SEIR compartmental model here we consider a purely temporal Hawkes process (Hawkes, 1971) here, where λ*(t)* is written as:

$$\lambda (t)=\mu +\text{K}\Sigma_{i:t_{i}<t}g\left(t-t_{i}\right).$$

**Fig. 1.**
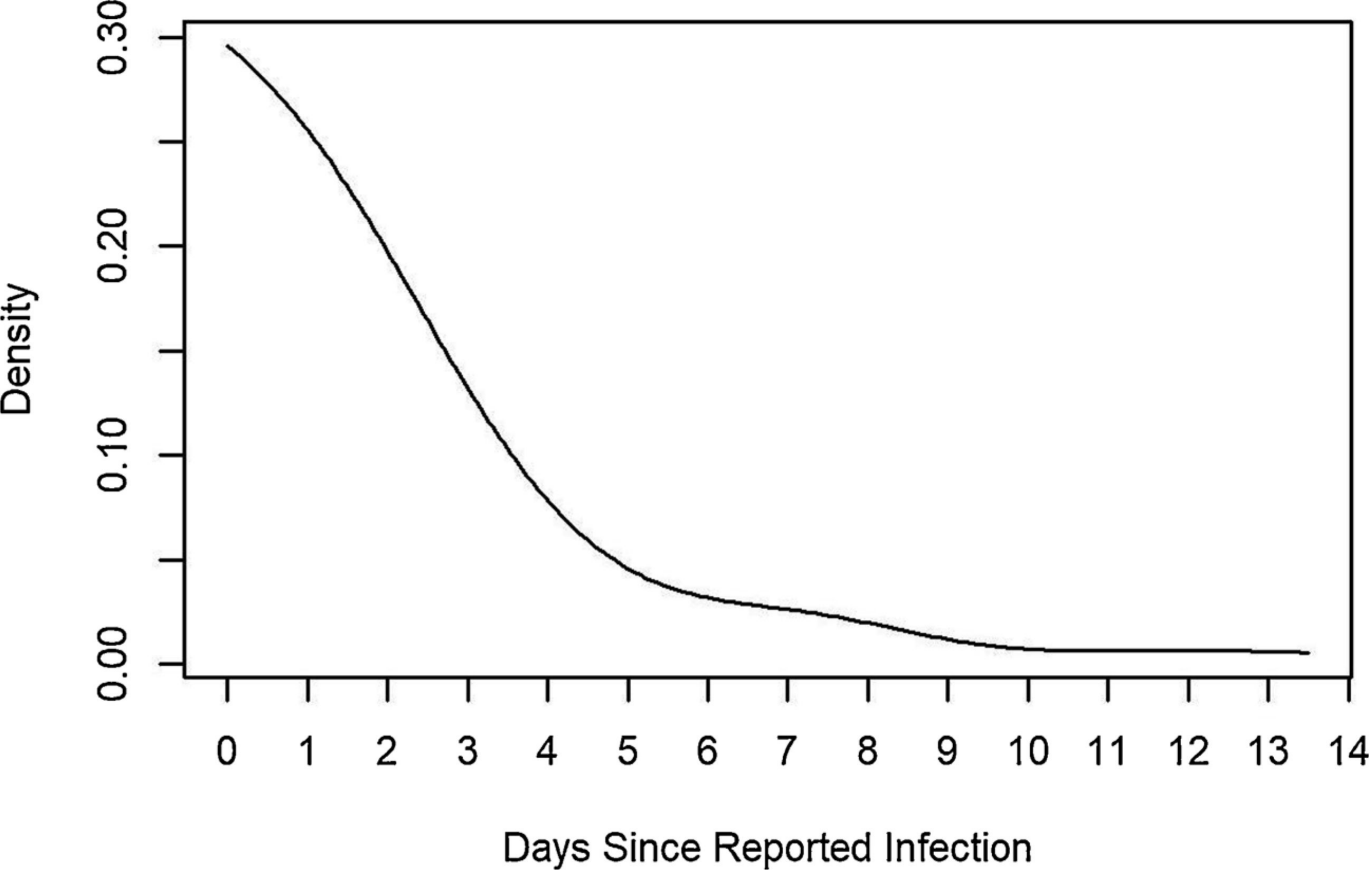
Fitted triggering density (smoothed) for the 2018–2019 EVD outbreak from May 3, 2018 to June 16, 2019. The x-axis represents days since infection as reported by WHO, where infection day was in some cases estimated based on how long patients were symptomatic.

The Hawkes model is estimated essentially by fitting a step function to the triggering density g, where the step heights and background rate *μ* are estimated by maximum likelihood, according to the method of Marsan and Lengliné (2008), and the step function is subsequently smoothed using a Gaussian kernel. The triggering density *g* indicates the rate at which infection is spread, and the fitted triggering density shows most secondary infections occurring within a week (Fig. 1).

The log-likelihood of an observed sequence of infections according to an estimated Hawkes model is:

$$l(\hat{\theta })=\Sigma_{i}\log\left[\lambda \left(t_{i};\;\hat{\theta }\right)\right]-\int_{0}^{T}\lambda (t;\;\hat{\theta })dt.$$

Here, $\hat{\theta }=(,)$ is the vector of parameter estimates. The log-likelihood can be computed on the data used to estimate the parameters or can be computed on data outside of the training sample. The log-likelihood is a measure of fit and is closely related to the entropy or information gain of the estimated model relative to a stationary Poisson model (Harte and Vere-Jones, 2005).

One application of the Hawkes model is to enable real-time forecasting of an EVD outbreak. Using the median of 1000 simulations of the fitted Hawkes model, we predicted the number of cases expected to occur over a nine-, six-, and three-week period, starting on April 14, 2019, May 5, 2019 and May 26, 2019, all ending on June 16, 2019, where each subsequent forecast uses model parameters re-estimated with updated data. Then using data up to June 16, we generated probabilistic projections of three-, six-, and nine- weeks based on prior research showing the degradation of epidemic forecasting accuracy over the long term (Worden et al., 2018; Chowell et al., 2017). We evaluated the accuracy of our probabilistic projections by comparing projected vs. actual outbreak sizes, the log-likelihood (information) score (Brocker and Smith, 2005) and the error per day in the median forecast. On April 14, 2019, May 5, 2019 and May 26, 2019 there were 1312, 1667 and 1956 reported EVD cases, respectively. We conducted all analyses using R 3.4.2 (R Foundation for Statistical Computing, Vienna, Austria).

## 3. Results

**Fig. 2.**
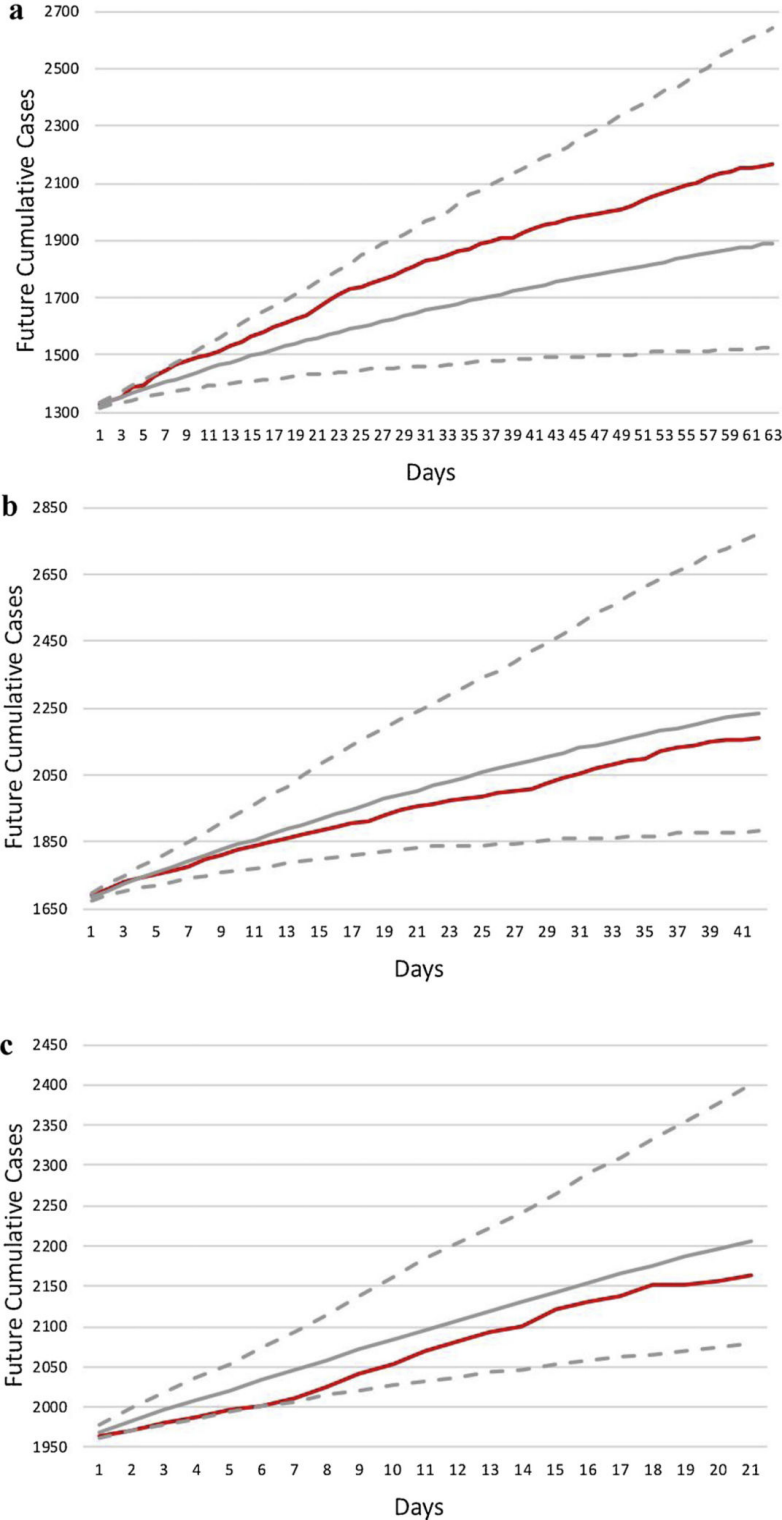
Median estimate of projected cumulative case counts (grey line) from April 14, 2019 (Fig. 2a), May 5, 2019 (Fig. 2b) and May 26, 2019 (Fig. 2c), all ending June 16, 2019, and the 95% prediction interval (dotted). Actual cumulative case counts are plotted for comparison (red line) but were not known at the time projections were made. (For interpretation of the references to colour in this figure legend, the reader is referred to the web version of this article.)

As of June 16, 2019, there were 2136 reported EVD cases across 22 health zones in the provinces of North Kivu and Ituri, DRC. Of these EVD cases, about 95.7% were confirmed and 4.3% were probable. We used the Hawkes model to generate nine-, six- and three-week probabilistic forecasts (all ending June 16, 2019) (Fig. 2). The median simulated outbreak size on June 16 was 1892 (95% prediction interval [PI]: 1525, 2641), 2236 (95% PI: 1881, 2773) and 2206 (95% PI: 2079, 2401) respectively. The errors in the median forecasts for the nine-, six-, and three-week forecasts were respectively 6.73 cases, 4.96 cases, and 4.85 cases per day. The log-likelihood (per case) evaluated on the data after the forecasts were made for the nine-, six- and three-week forecasts were 1.60, 1.43 and 1.24, respectively. The higher log-likelihood per day for the 9-week forecasts appears to be attributable to the increased number of observed cases during the first few weeks of the forecasting period causing a sharp increase in the sum of log(λ) term in the log-likelihood.

**Fig. 3.**
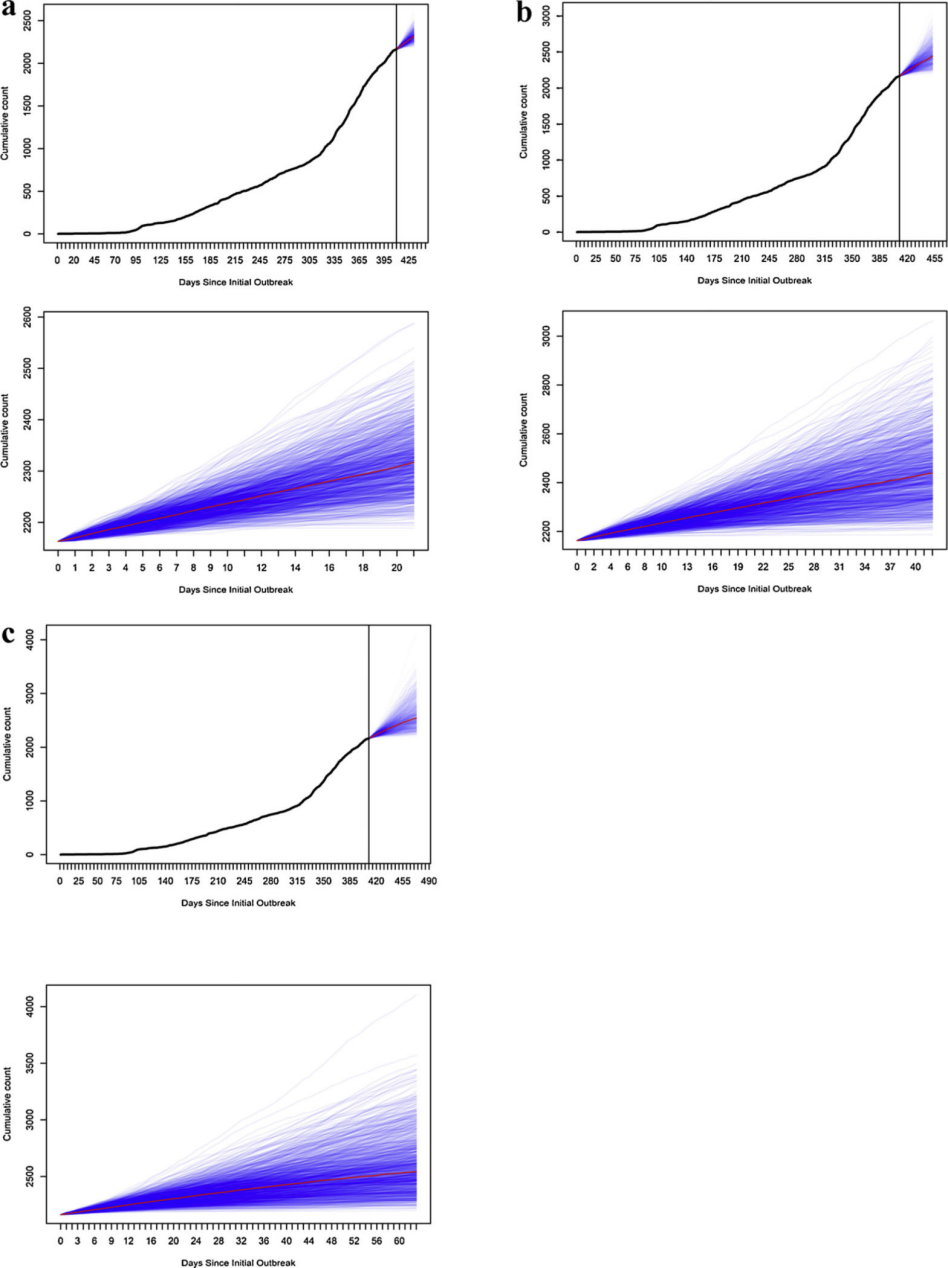
Epidemic curve, as of June 16 (cutoff at vertical line), followed by three- (Fig. 3a), six- (Fig. 3b), and nine-week (Fig. 3c) probabilistic projections (blue lines) of case counts, using the Hawkes model (median, red line), both with outbreak history and zoomed-in. (For interpretation of the references to colour in this figure legend, the reader is referred to the web version of this article.)

In our forecast of the unobserved period using data up to June 16, the three-, six-, and nine-week probabilistic projections of median outbreak size were respectively as follows: 2317 (95% PI: 2222, 2464); 2440 (95% PI: 2250, 2790); and 2544 (95% PI: 2273, 3205) (Fig. 3). The log-likelihood score (per case) of the estimated Hawkes models in Figs. 2a–c and 3 are 0.69, 0.93, 1.04 and 1.06, respectively. Projected and actual outbreak sizes followed a near linear increase (Fig. 2).

## 4. Discussion

We employed a non-parametrically estimated Hawkes point process model to generate multiple probabilistic projections of the ongoing 2018–2019 EVD outbreak size in DRC. As seen in Fig. 2, the median nine-week projection experienced some degradation with forecast errors of 6.73 cases per day, whereas the six- and three-week projections were more reliable, with errors in the median forecasts of 4.96 and 4.85 cases per day, respectively, and with the observed number of cases falling well within the estimated 95% prediction window obtained using simulations of the fitted Hawkes model for the three-week period. These findings were consistent with other modeling studies that have shown how even short-term forecasts can degrade over longer periods of time (Funk et al., 2018; Worden et al., 2018). Our results support earlier work performed using Hawkes point process models to predict the size of infectious disease outbreaks; our models of the 2013–2016 EVD outbreak in West Africa reduced root mean squared error (RMSE) by as much as 38% when compared to traditional compartmental models (Park et al., 2018). Growing evidence, including the work presented here, suggests that point process models can provide accurate estimates of caseloads for a wide variety of epidemics, including both ongoing and previous Ebola outbreaks (Schoenberg et al., 2017; Park et al., 2018).

The Hawkes model performed well during this outbreak with minimal modeling assumptions, and could be a valuable tool for real-time decision making amidst ongoing outbreak of EVD or other diseases. In its non-parametric form, a disadvantage of the Hawkes model may be its inability to parameterize contexts that may help explain the current epidemic trajectory. While these factors (e.g., contact tracing and clinical care) may be considered in future iterations of the Hawkes model (Funk et al., 2017), developing these parameters can also delay model development and application. Even with such parameters estimated, some factors in real biological epidemics, such as political unrest or armed conflict that affect disease transmission rates, can be challenging to parameterize in statistical models.

While the Hawkes model’s simplicity has advantages, it can also be viewed as a limitation when, for instance, inhomogeneity of the background rate or changes in productivity lead to overestimation of finescale clustering, leading to a triggering function estimate that may be less biologically plausible. Unanticipated shocks (e.g., introduction of EVD into a large metropolitan area) that occur after predictions may decrease the model’s accuracy beyond our uncertainty estimates. In addition, these models estimate future cases via the triggering function, which requires scrutiny due to its tendency to underestimate secondary transmission rates. Should dynamics of the disease rapidly change at a time period for which data was not included (e.g. driving productivity to a value greater than 1), a relatively simple point process model may not account for such rapid shifts in triggering and may not be able to anticipate them in forecasts. While the Hawkes model might be able to adjust to decreasing numbers of cases as data become available and parameter estimates change, it may well be that the Hawkes model fails to perform well as the disease cases wane near the end of an outbreak, and this behavior should be a major subject of future research in assessing the forecasts made here.

As such, we do not suggest here that these models replace traditional compartmental models (SIR and their relatives). Rather, we see these models as complementary, and in the particular case of requiring rapid response and prediction of caseloads, a valuable addition for efforts that attempt to limit the impacts of an outbreak. In an effort to continue to evaluate the efficacy of these models in predicting outbreak rates and cumulative cases in real-time (or near real-time, given the time it takes for corrected caseload data to be released), we have constructed a free, publicly-accessible website that can track this and other outbreaks, with purely prospective forecasts and results updated weekly as new data become available (for full details, see http://www.stat.ucla.edu/~frederic/ebola).

In conclusion, we are encouraged by the ability of non-parametric Hawkes point process models to describe epidemic events over the short term and in real time that are consistent with the 2018–2019 EVD outbreak in DRC. The Hawkes point process is a relatively simple statistical model, and results suggest that statistical modelers in the public health community should consider the Hawkes model in their ensemble when engaging in decision-making and resource allocation of EVD and other emerging infectious disease outbreaks.

## Supplementary Material

supplemental material
